# *Krüppel-homologue 1* regulates the development of *Tuta absoluta* and its cascade regulation pattern in the juvenile hormone signalling pathway

**DOI:** 10.1098/rsob.220372

**Published:** 2023-05-31

**Authors:** Xiaodi Wang, Siyan Bi, Yanhong Tang, Guifen Zhang, Cong Huang, Fanghao Wan, Zhichuang Lü, Wanxue Liu

**Affiliations:** ^1^ State Key Laboratory for Biology of Plant Diseases and Insect Pests, Institute of Plant Protection, Chinese Academy of Agricultural Sciences, Beijing 100193, People's Republic of China; ^2^ Agricultural Genome Institute at Shenzhen, Chinese Academy of Agricultural Sciences, Shenzhen 518120, People's Republic of China

**Keywords:** *Tuta absoluta*, juvenile hormones, *takr-h1*, cascade regulation

## Abstract

Tomato leaf miner, *Tuta absoluta* (Meyrick), is one of the most destructive quarantine pests globally. It has been confirmed that *Krüppel-homologue 1* (*kr-h1*) plays a key role in the regulation of juvenile hormone (JH). However, it is unclear how *kr-h1* regulates the synthesis of JH and its cascade regulation pattern in tomato leaf miner. Here, we obtained the six JH signalling genes (*kr-h1*, *Methoprene-tolerant*, *Forkhead box O*, *Juvenile acid methyltransferase*, *Juvenile hormone esterase* and *Fatty acid synthase 2*), and applied RNA interference to explore the role of *kr-h1* and the seven genes (plus *Vitellogenin*) regulation relationship in *T. absoluta*. Bioinformatics analysis revealed the structural characteristics of kr-h1 protein and JH receptor Met, which contained eight C2H2 zinc finger structures and three typical domains of the bHLH-PAS family, respectively. The expression levels of *Met* and *Vg* were upregulated after RNAi of *kr-h1* gene, while the gene levels of *JHAMT* and *FAS2* were downregulated. Furthermore, topical application of JH analogue to second instar larvae could induce the expression of *kr-h1* and inhibit the expression of *Met*. Our study reveals the mechanism by which *kr-h1* regulates JH pathway genes, which could be applied to control the growth of tomato leaf miners.

## Graphical abstract

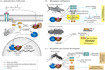

## 1. Introduction

Native to Peru and Western South America, tomato leaf miner, *Tuta absoluta*, is a known worldwide quarantine pest. Since 2006, it has been introduced to Spain and Europe, and has spread to Africa and Asia [1–5]. In China, this invasive species was first discovered in August 2017 in Huocheng County, Ili Kazakg Autonomous Prefecture, of the Xinjiang Uygur Autonomous Region (Huocheng, Ili, Xinjiang) [6]. Therefore, effective prevention and control measures should be taken in these areas, as well as in areas where tomato leaf miner has not yet spread. Considering its insect selectivity and gene specificity, RNA interference (RNAi) technology is the most feasible bioengineering technology applied in pest control, and has shown good application potential in gene therapy and genetic control of pests [7–10]. In-depth analysis of key genes regulating insect growth, development, reproduction and behaviour, and making full use of pest-specific genes and gene sequences, are key to RNAi success in pest control strategies. The key insect functional genes are generally classified into several categories. The first is juvenile hormone (JH) or ecdysone signalling pathway genes, which include *Met*, *Gce*, *kr-h1*, *EcR*, *USP*, *Br*, etc. Interference with these genes could lead to phenotypes such as early pupation, moulting defects or direct death in *Drosophila melanogaster*, *Spodoptera exigua*, *Blattella germanica*, *Helicoverpa armigera*, *Bombyx mori*, *Sitobion avanae*, *Grapholita molesta*, *Locusta migratoria*, *Tribolium castaneum* and *Spodoptera litura* [11–23]. The second category comprises reproductive- and oviposition-related signalling pathway genes, such as hydroxy-3-methylglutaryl CoA of *H. armigera* [24], vitellogenin receptor of *S. litura* [25], epidermal protein gene of *Myzus persicae* [26], ATP hydrolase and cyclophilin B gene of *Bemisia tabaci* [27,28]. The final group is immunity- and resistance-related genes, such as small G protein *Ras* gene of *S. exigua* [29], the cytochrome P450 *CYP6AE14*, *CYP6BG1*, *CYP321A8*, *CYP321B1* and *CYP6AE44* genes of *H. armigera* [30–33].

**Figure 1.**
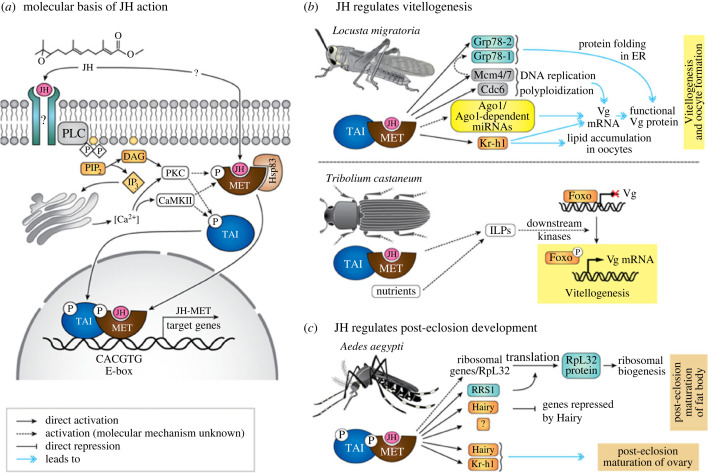
Relationship of juvenile hormone signalling pathway [34].

This study mainly focuses on JH, a unique insect hormone that is involved in moulting, reproduction, mating and behavioural regulation of insects, which is considered one of the most important hormones for insect life (figure 1) [34,35]. Furthermore, JHs are evolutionarily conserved in insects with both complete and incomplete metamorphosis [35–38]. Thus, JHs signalling pathway can provide targets for the synthesis of pest control agents. In 2013, Jindra *et al*. [35] identified that methoprene-tolerant (*Met*) is the nuclear receptor of JH, suggesting that JHs primarily exert their effects on gene expression and function within the nucleus, thereby gradually revealing the molecular mechanism of JH. Further research found that *Met* has a paralogous gene germ cell-expressed (*Gce*). Gce is also a bHLH-PAS (basic helix-loop-helix Per-AhR/Arnt-Sim) protein that can bind to JH [39,40]. Gce exists in *D. melanogaster*, when the concentration of JH in the cytoplasm is low, so that Met polymerises into homodimer Met-Met or heterodimer Met-Gce. When the concentration of JH in the cytoplasm is high, JH binds to Met, leading to Met-Met/Met-Gce dissociation [39,41,42], followed by Met binding to steroid receptor coactivator (SRC) or FISC (*β*Ftz-F1 interacting steroid receptor coactivator) to form transcription complex Met/SRC or Met/FISC and bind to the JHRR region response element of JH, which regulates the expression of kr-h1 [36,43–45]. As a key downstream response protein in the JH signalling pathway, kr-h1 encodes a transcription factor containing a C2H2 zinc finger structure that regulates the synthesis of JH. Forkhead box O (FOXO) is a transcription factor downstream of the phosphatidylinositol 3-kinase/protein kinase B (PI3K/Akt) pathway within the insulin signalling pathway, and its activity is inhibited by the PI3K/Akt pathway [46]. In a study with *Culex pipiens*, JH decreased FOXO expression, thereby inhibiting lipid accumulation in females [47]. Juvenile hormone esterase (JHE) is a metabolic enzyme whose synthesis promotes JH hydrolysis, thereby reducing JH titre during certain periods of insect development and thus regulating metamorphosis. The impact of JH on important physiological processes means its titre must be strictly controlled. Specifically, juvenile acid methyltransferase (JHAMT) has been identified as one of the most decisive enzymes regulating JH synthesis [48–50]. JHAMT plays a role in the last step of the insect JH biosynthesis pathway, during which it can activate and convert JH acid and inactive JH precursor into active JH [51]. Fatty acid synthase (FAS) catalyses the synthesis of acetyl-CoA, malonyl-CoA and NADPH into long-chain saturated Fatty acids [52,53], thereby regulating lipid accumulation during diapause in insects [54]. Vitellogenin (Vg) is a precursor protein of vitelloprotein, which provides energy reserves for embryonic development [55,56]. The expression patterns of these genes are dynamically regulated throughout insect growth and development.

To further explore the functions of genes related to the JH pathway, this study depicted the expression profiles of *kr-h1*, *Met*, *FOXO*, *JHAMT*, *JHE*, *FAS2* and *Vg* at different developmental stages, and then cloned the full-length coding regions of these six genes. The nucleotide and amino acid sequences of *kr-h1* and *Met* in particular were analysed in detail. The role of *kr-h1* in the regulation of growth and development of tomato leaf miner and the regulatory relationship of these genes were explored. Therefore, in this study, we identified pest control target genes by screening the JH pathway genes, revealed their regulatory mechanisms, and then suggested ways in which these targets could be applied to control unwanted growth of tomato leaf miners.

## 2. Material and methods

### 2.1. Insects and treatments

The tomato leaf miners used in this experiment were collected in August 2018 within Yuxi, Yunnan Province, and kept in the laboratory at a temperature of 25 ± 2°C, relative humidity of 50–60%, and light cycle of 14 : 10 h (light : dark). The host plant, *Lycopersicum esculentum* Mill, was planted alone in 9 cm diameter pots in the same environment as the tomato leaf miner.

Eggs, larvae at different developmental stages, pupae, adults, RNAi and JHA-treated larvae were collected and placed in Eppendorf tubes and labelled. Then all samples were immediately frozen with liquid nitrogen for 60 s and stored at −80°C.

### 2.2. RNA extraction and real-time quantitative PCR (RT-qPCR)

Total RNA was extracted from the collected larvae, and first-strand cDNA was synthesized according to the manufacturer's instructions (TransGen Co. Beijing, China). Quantitative primers were designed by Primer 5 software based on the transcriptome database of *T. absoluta*. Primers were listed in table 1, and *Rpl5* was used as the internal reference gene for relative quantification. Relative expression levels of JH pathway genes were amplified by PCR, and amplification reactions were conducted in 20 µl containing 8.2 µl ddH_2_O, 10 µl qPCR SYBR Green Master Mix, 0.4 µl Forward Primer (10 µM), 0.4 µl Reverse Primer (10 µM) and 1 µl DNA template. The PCR cycling conditions were as follows: cycling stage (40 cycles): 95°C 5 min, 95°C 10 s, 60°C 30 s; melt curve stage: 95°C 15 s, 60°C 1 min, 95°C 30 s, 60°C 15 s. The relative expression levels were calculated using the comparative Ct (2^−ΔΔCt^) method [57,58]. All the experiments were performed in triplicate.

**Table 1.** Primers for real-time quantitative PCR.

gene name	primer name	sequence (5'-3′)	length (bp)
*kr-h1*	qkr-h1-F	CTGGTGAACGACCTTTTGAATG	22
	qkr-h1-R	GGTATGGTTTTTCTCCTGTGTGTG	24
*Met*	qMet-F	TGGTCCGATTCATTTCTCAA	20
	qMet-R	GCAATAGCCCAGTTTCTCCT	20
*FOXO*	qFOXO-F	GAAATGGAGAGGCAGGGCTGATGTG	25
	qFOXO-R	CGCTGTGTAGTGGTGAGTCTGGGAA	25
*JHAMT*	qJHAMT-F	GGACCGCTTCATCTCTCCCTACC	23
	qJHAMT-R	ATTCACCGCTGACACCGCATTTT	23
*JHE*	qJHE-F	CCGTCATCGAGTCCCCTCT	19
	qJHE-R	TCGGAAACCTCTTTAGCCC	19
*FAS2*	qFAS2-F	CTGCCTCTGGTCTTTGCTCTA	21
	qFAS2-R	GCTCCACTCATTCCACGGTTC	21
*Vg*	qVg-F	GCGGAACTTCTACTTCAGCGACTC	24
	qVg-R	TAGTGGCGGTCTTGATGTTCTTGC	24
*Rpl5*	qRpl5-F	CAGTCGTCGAGCCAGCAACA	20
	qRpl5-R	TCCCGCATTGAAGGAGACCA	20

### 2.3. Gene cloning

We found the sequences for the six genes *kr-h1*, *Met*, *FOXO*, *JHAMT*, *JHE* and *FAS2* of *Adoxophyes honmai*, *A. aegypti*, *Aphis glycines, B. dorsalis*, *D. melanogaster*, *B. mori* and *Culex quinquefasciatus* in NCBI (GenBank). The nucleotide sequences were compared with the transcriptome of tomato leaf miner (using BLASTN to obtain the corresponding sequence of tomato leaf miner). Based on this information, specific primers were designed and these sequences were cloned by RACE. The sequences of the forward primers and reverse primers are listed in table 2.

**Table 2.** Primers for full-length gene amplification.

gene name	primer name	sequence (5'-3′)	length (bp)
*kr-h1*	kr-h1-F	TCAACAAATTCCGCATTCG	19
	kr-h1-R	TTTCAGGCAACATTCAACG	19
*Met*	Met-F1	AGTGAACTTGTCCAGTGAGC	20
	Met-R1	CAATAGCCCAGTTTCTCC	18
	Met-F2	GCGTCTTATTCGCAGTC	17
	Met-R2	GGTTTATGAGTGTTGGGTGT	20
*FOXO*	FOXO- F1	GCCCAGACCCGACAACTA	18
	FOXO R1	CGGAAGACGACGACAACG	18
	FOXO-F2	CTCGCTGACTCCTTGAAAC	19
	FOXO-R2	CACTAAAGCCCTACAACTAACA	23
*JHAMT*	JHAMT-F1	CGAATTTGCTGCAAGCG	17
	JHAMT-R1	CGTCAAACAGCGGCATG	17
	JHAMT-F2	ACATCTACGACCTGTTGGC	19
	JHAMT-R2	GGGATAAATATGGCGTTCT	19
*JHE*	JHE-F1	AGTCAGATAACCGGCGAGAT	20
	JHE-R1	GCGTCAGGGTCTGTAGCA	18
	JHE-F2	TCCCTGAACACGACTAAG	18
	JHE-R2	TGTCCGCAAATCAATCT	17
*FAS2*	FAS2-F1	TTCGAGTCCCGAATAAAGT	19
	FAS2-R1	GTGGAGGAGTTGTGCGTAT	19
	FAS2-F2	TCGGTATCACTGGATGCTC	19
	FAS2-R2	AGGCTGCTACTCCTTCACG	19
	FAS2-F3	AGTCGGAGACCCAGAAGA	18
	FAS2-R3	TTTGGAATGGTACGCAAT	18
	FAS2-F4	TAAGACTATCTGCCCTCCG	19
	FAS2-R4	GTAACCCAGTTGTTGACCC	19
	FAS2-F5	CGAGGCGTCATTGGTTGTA	19
	FAS2-R5	TCAGGGTTGAAAGGTGGTG	19
	FAS2-F6	CGAACAAGTCAAGGAGGAG	19
	FAS2-R6	TAGGCACAGCATCAATAGGC	20
	FAS2-F7	TCGGAAAGGTCATCGTCAA	19
	FAS2-R7	CCTCATCAGCACTCGGAAA	19

### 2.4. Bioinformatics analysis

The Open Reading Frame (ORFs) Finder (https://www.ncbi.nlm.nih.gov/orffinder) was used to check the ORFs. The full-length coding regions of *kr-h1* and *Met* were obtained by sequence translation and splicing using DNAMAN software. SMART (http://smart.embl-heidelberg.de/) software was used to predict the conserved protein domains. SWISS-MODEL (https://swissmodel.expasy.org/) was used to predict the tertiary structure of protein. Multiple alignment of protein sequences was performed by ClustaW software. Phylogenetic trees were constructed using the maximum-likelihood method in MEGAX software.

### 2.5. RNA interference

To generate dsRNA, three fragment templates of *Takr-h1, TaMet* and *TaFOXO* were amplified by PCR using cDNAs cloned previously as templates with forward and reverse primers containing the T7 primer sequence (table 3) at the 5′ ends, respectively. Amplification reactions were conducted in 25 µl solutions with 19.0 µl of ddH_2_O, 2.5 µl of 10 × buffer, 0.5 µl of dNTPs (10 mM for each nucleotide), 1.0 ml of forward primer (10 mM µl^−1^), 1.0 ml of reverse primer (10 mM µl^−1^), 0.5 µl of cDNA template and 0.5 µl of Taq DNA Polymerase (5 Uµl^−1^; TransStart). The PCR cycling conditions were as follows: 94°C for 5 min, followed by 35 cycles of 94°C for 30 s, 60°C for 30 s and 72°C for 30 s, and a final extension step of 72°C for 10 min. The amplification of the PCR products was confirmed by separation on 1.5% agarose gels and visualized by staining with ethidium bromide under ultraviolet (UV) light. The sequencing was verified at Sangon Biotech. The dsRNA was synthesized using a MEGAscript RNAi Kit (Ambion, Austin, TX, USA). One µg of PCR product was used as a transcription template. The dsRNA was resuspended in RNase free water. The dsRNA was analysed by agarose gel electrophoresis and quantified spectrophotometrically. The dsRNA was stored at −80°C until required. Detached leaflets (length: about 6 cm, width: about 4 cm) from tomatoes had their petioles immersed in 200 µl of water containing either 5 µg of dsRNA from each target gene or a GFP control (each in triplicate). Uptake of the dsRNA solution by the tomato leaflets took 3–4 h. Immediately after uptake, second instar larvae (*n* = 15) were gently placed onto leaflets for feeding, and individuals were sampled 48 h after initiation of feeding.

**Table 3.** Primers for dsRNA synthesis.

GENE name	primer name	sequence (5'–3')	length (bp)
*kr-h1*	dskr-h1-F	taatacgactcactatagggTGGTTGCGGTAAAGGATT	38
	dskr-h1-R	taatacgactcactatagggTAACAGGGTCTGGCTGAG	38
*Met*	dsMet-F	taatacgactcactatagggGACCTCAGCATCTCCTTA	38
	dsMet-R	taatacgactcactatagggTACTCCACTGTGCCTTTT	38
*FOXO*	dsFOXO-F	taatacgactcactatagggCCACTACACAGCGGCTTTCAG	41
	dsFOXO-R	taatacgactcactatagggCCATCATCCCACCGTTCATCA	41
*EGFP*	dsEGFP-F	taatacgactcactatagggTGAGCAAGGGCGAGGAG	37
	dsEGFP-R	taatacgactcactatagggCGGCGGTCACGAACTCCAG	39

### 2.6. JHA treatment

JHA methoprene (CAS-number: 40596-69-8; MedChemExpress, China) was diluted to 25 mg ml^−1^ concentrations using dimethyl sulfoxide (DMSO) and stored at −80°C as a stock solution. For a working solution, the JHA methoprene was diluted to 2.5 µg µl^−1^ with normal saline. A DMSO solution diluted with normal saline was used as a negative control. For treatment, 1 µl of control solution or treatment liquor was dropped onto the back of the larva using a micro syringe.

### 2.7. Statistical analysis

Statistical analysis was performed using Student's *t*-test and ANOVA analysis. Data are presented as mean ± s.e.m. of three independent biological replicates, and *p* < 0.05 was considered as significant. Different letters above the columns indicate significance in the group difference and significance levels are indicated with asterisks: *p < 0.05, **p < 0.01 and ***p < 0.001.

## 3. Results

### 3.1. Expression of *kr-h1*, *Met*, *FOXO*, *JHAMT*, *JHE, FAS2* and *Vg* during different developmental stages

**Figure 2.**
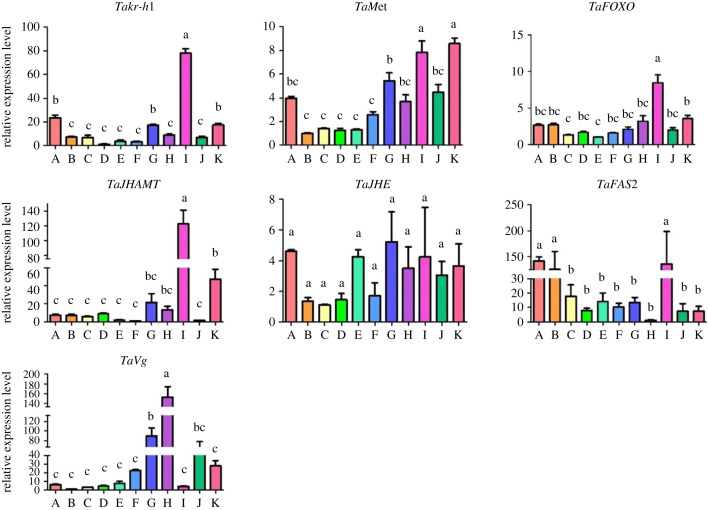
Expression profiles of seven JH signalling-related genes at different developmental stages of *T. absoluta.* The horizontal axis represents different developmental stages, A to K representing the first instar larval stage (2 d), the second instar larval stage (4 d), the third instar larval stage (6 d), the fourth instar larval stage (8 d), early pupation (14 d), late pupation (18 d), early female (21 d), early male (21 d), late female (30 d), late male (30 d), respectively. The vertical axis shows the relative expression level is expressed by mean ± s.e.m. The different letters (a, b) represent groups with significant differences according ANOVA test (Tukey's test, *p* < 0.05).

The expression profiles of the various JH signalling pathway genes during different developmental stages were determined by real-time quantitative PCR (RT-qPCR) (figure 2). The study found that the expression levels of the JH signalling pathway genes were different depending on the developmental stage. For instance, mRNA expression of *Takr-h1* in newly emerged males was the highest in all developmental stages, and the *Takr-h1* mRNA expression levels in the egg, late pupal, and mature stages in male adults were significantly higher than those in larvae and female adults. The expression of *TaMet* did not significantly differ from egg to early pupation, although it was appreciably higher in males than in females. The mRNA expression levels of *TaFOXO* and *TaJHAMT* in newly emerged males were significantly higher than *T. absoluta* in other stages. There were no significant differences in the expression levels of *TaJHE* between each instar stage. The relative levels of *TaFAS2* mRNA expression during the egg, first instar larvae, and newly emerged male stages were significantly higher than those in other stages. Unlike the above genes, the relative expression of *TaVg* was significantly higher in newly emerged females compared with other periods. Additionally, the relative expression levels of *kr-h1* mRNA were also high. Considering *kr-h1* acts as a key mediator of JH signalling and has been extensively studied in other insects, we chose to first look at the *kr-h1* gene in our in-depth study of the JH pathway.

### 3.2. Cloning and sequence analysis of genes related to JH signalling pathway

The complete ORF sequences of *TaKr-h1*, *TaMet*, *TaFOXO*, *TaJHAMT*, *TaJHE* and *TaFAS2* were cloned using PCR. Among them, the ORF sequence of *kr-h1* was 1044 bp in length, the 5'-untranslated region (5'-UTR) was 135 bp, and the 3'-UTR was 90 bp, encoding 347 amino acids (electronic supplementary material, figure S1*a*). The molecular weight (MW) of TaKr-h1 was 38.80 kDa, and the theoretical isoelectric point (pI) was 9.24. The ORF sequence of *Met* was 1521 bp in length, the 5'-UTR was 118 bp and the 3'-UTR was 275 bp, encoding 506 amino acids (electronic supplementary material, figure S1*b*). The MW of TaMet was 58.16 kDa, with a theoretical isoelectric point of 8.32. The general information for the other four genes identified from *T. absoluta* is listed in table 4.

**Table 4.** General information of genes identified from *Tuta absoluta*.

genes	ORF length (bp)	5'-UTR	3'-UTR	number of amino acids	molecular weight (kDa)	isoelectric point
*Kr-h1*	1044	135	90	347	38.80	9.24
*Met*	1521	118	275	506	58.16	8.32
*FOXO*	1167	226	255	388	41.87	5.43
*JHAMT*	1143	22	139	380	43.91	8.75
*JHE*	1758	18	310	585	64.35	6.37
*FAS2*	7260	52	107	2419	267.53	6.18

The structure of *Takr-h1* was analysed using SMART online software. Its structure included eight C2H2 zinc finger structures (electronic supplementary material, figure S2*a*), each containing two cysteines (Cys) and two histidines (His). Notably, most the proteins with zinc finger structure are functional proteins related to the regulation of gene expression, in which they promote the binding of DNA or RNA. This structure has thus been highly conserved throughout biological evolution. Met protein has four structures (electronic supplementary material, figure S2*b*). The first is a HLH (helix-loop-helix), which comprises a short α-helix and another long α-helix through a loop, enabling it to bind to corresponding gene fragments and play a regulatory role in gene transcription. The next two structures within Met are PAS domains, which are involved in many signalling protein pathways and used as signal sensor domains. The last component of Met, the PAC domain, is closely related to the PAS domain. The tertiary structures of kr-h1 and Met were predicted respectively, and their conserved domains were marked accordingly (electronic supplementary material, figure S2*c,d*). Multiple sequence comparisons with other insect species showed that the amino acid sequences of kr-h1 and Met were 85.27% (electronic supplementary material, figure S2*e*) and 84.56% (electronic supplementary material, figure S2*f*) similar to proteins in other species, respectively, indicating that these two genes are highly conserved in insects. Phylogenetic analysis revealed that *Takr-h1* is clustered with *kr-h1* in the insects *Spodoptera exigua*, *H. armigera*, *G. molesta*, *B. mori*, thereby forming an orthologous group for *kr-h1* (electronic supplementary material, figure S2*g*). The results of Met protein clustering showed that the *T. absoluta* was clustered with the species *S. frugiperda*, *H. armigera*, *Agrotis ypsilon*, *Mythimna separata*, *B. mori*, which was consistent with traditional taxonomy (electronic supplementary material, figure S2*h*).

### 3.3. *Takr-h1* function identification in the growth and development of *T. absoluta*

**Figure 3.**
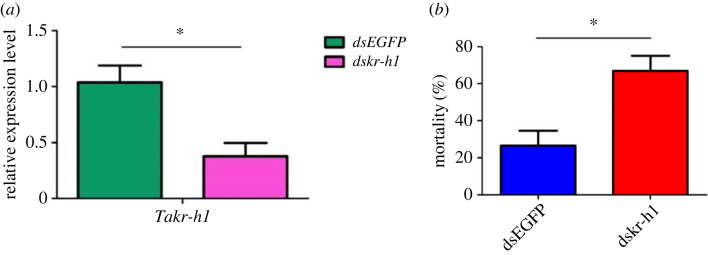
Changes in the relative expression of *Takr-h1* gene and mortality of *T. absoluta* after feeding *dskr-h1.* (*a*) Changes in the relative expression of *Takr-h1* gene*.* (*b*) Mortality of *T. absoluta* after feeding *dskr-h1.* The blue column indicates feeding *dsEGFP*, and the red column indicates feeding *dskr-h1*. Data are presented as mean ± s.e.m.; on the horizontal line represents the difference of relative gene expression under different treatment conditions (*p < 0.05).

**Figure 4.**
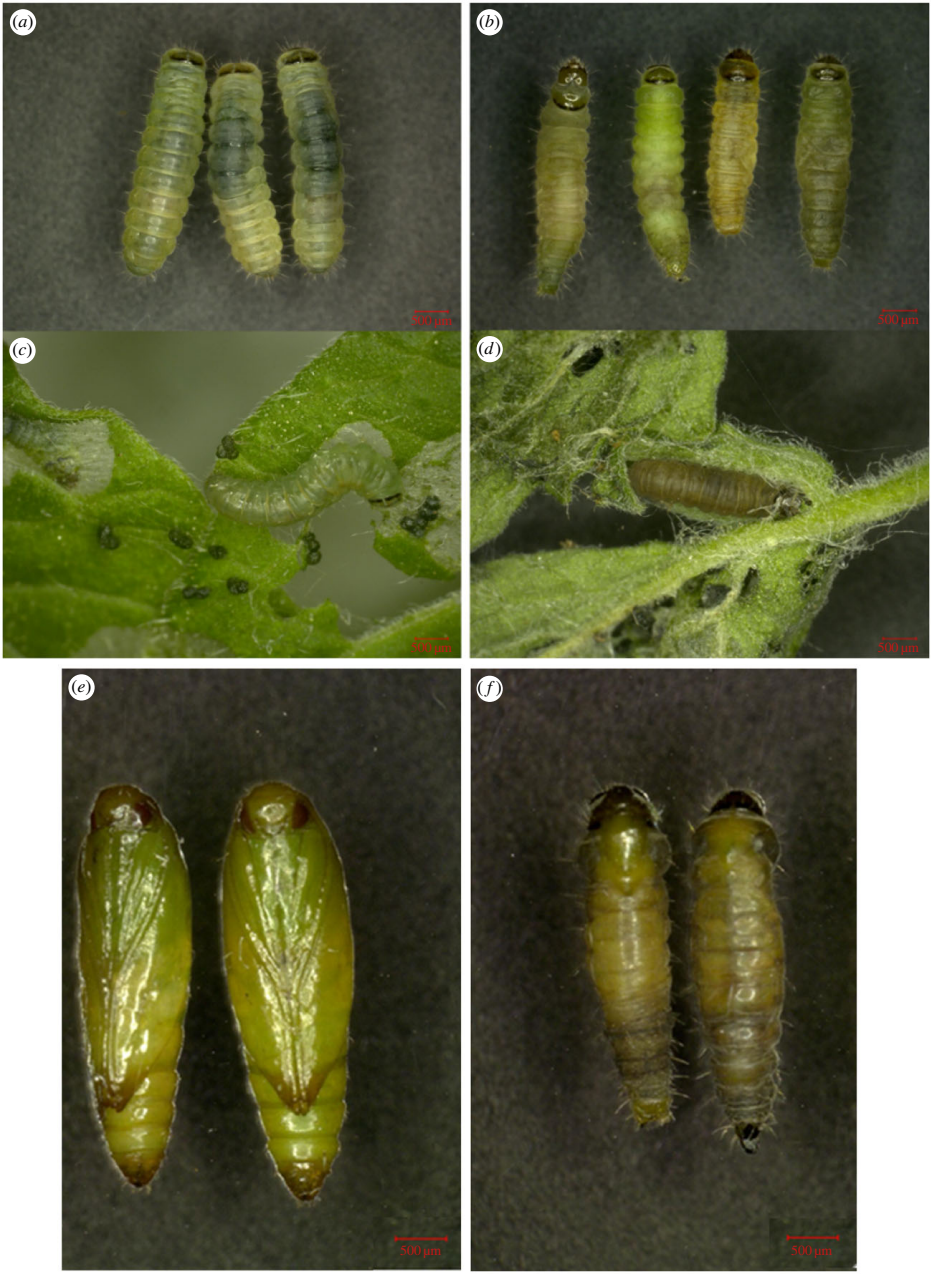
Effects of feeding *dskr-h1* on phenotype of *T. absoluta*. (*a*) Phenotype of *T. absoluta* larvae fed with *dsGEFP*. (*b*) Phenotype of *T. absoluta* larvae after feeding *dskr-h1*. (*c*) Feeding status of *T. absoluta* larvae after feeding *dsEGFP*. (*d*) The state of leaf death of *T. absoluta* larvae after feeding *dskr-h1*. (*e*) The phenotype of *T. absoluta* pupa after feeding *dsEGFP*. (*f*) The phenotype of *T. absoluta* pupa after feeding *dskr-h1*.

To investigate the function of *Takr-h1* during the growth and development of larvae, *Lycopersicum esculentum* Mill leaves with petiole were fed with *dskr-h1* dsRNA during the second instar. RT-qPCR analysis showed that the expression of *Takr-h1* decreased significantly (62.3%) after *dskr-h1* dsRNA treatment for 48 h (figure 3*a*). Furthermore, the mortality rate of tomato leaf miners increased significantly, reaching 66.87% (figure 3*b*). Phenotypic observation found that the normal growing larvae were body full (figure 4*a*) and actively feeding (figure 4*c*). Conversely, the epidermises of the dead larvae were shrunken, the body surfaces turned blacker in colour as time after death increased (figure 4*b*), and blackened larvae were found in the leaves (figure 4*d*). Moreover, deformities such as atrophy and wrinkling of the pupal skin were also observed (figure 4*f*). These observations indicate that the *Takr-h1* gene plays a key role in the growth and development of tomato leaf miner.

### 3.4. Cascade regulation between *Takr-h1* and other juvenile hormone pathway genes

**Figure 5.**
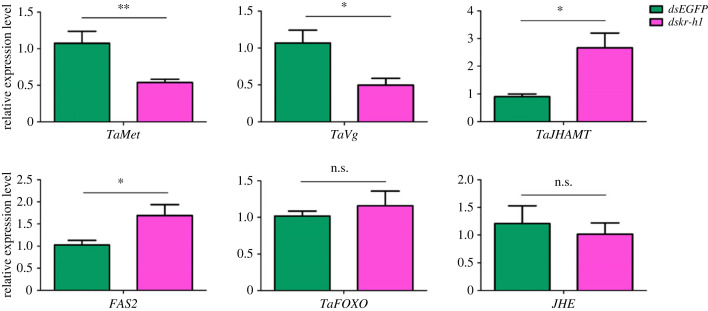
Effects of *Takr-h1* silencing on expression levels of JH signalling-related genes. The relative expression levels are expressed as the mean ± s.e.m. The green column represents the control group that was fed *dsEGFP*, while the pink column represents the treatment group that was fed *dskr-h1*. Asterisks represent significance levels: *p < 0.05, **p < 0.01; n.s. indicates no significance *p* > 0.05.

To further determine how *Takr-h1* regulates other transcription factors and receptors within the JH pathway, the expression of JH signalling pathway genes in second instar larvae of the tomato leaf miner was examined following treatment with feeding *dskr-h1.* Notably, RT-qPCR analysis showed that compared with the control group, silencing of *Takr-h1* significantly reduced the expression of *TaMet and TaVg*, while increasing the expression of *TaJHAMT* and *FAS2*. There were no significant differences in levels of *TaFOXO* and *TaJHE* expression (figure 5).

**Figure 6.**
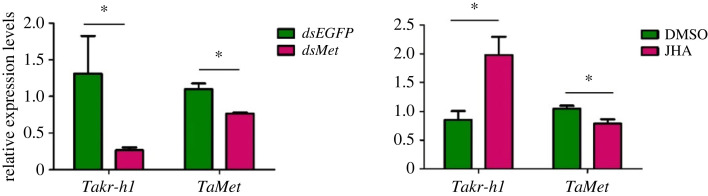
Changes of relative expression of three genes after feeding *dsMet* and JHA. The relative expression levels are expressed as the mean ±s.e.m.; the green column represents the control group and the red column represents the treatment group.Asterisk represents significance level:*p < 0.05.

To determine the regulatory relationship between *kr-h1* and *Met*, we measured the changes in expression of this two genes following treatment with *dsMet* or JHA (figure 6). The results showed that after *Met* RNAi, the mRNA expression levels of *Met* and *kr-h1* decreased by 23.04% and 73.33%, respectively, compared with the control. Meanwhile, tomato leaf miner treated with JH analogue JHA (methoprene) exhibited a significant increase in *Takr-h1* expression of 97.93%, whereas the expression of *TaMet* mRNA decreased by 20.88%. The above results indicate a mutual regulatory relationship exists between these two genes, such that they play a joint role in regulating the growth and development of tomato leaf miners.

**Figure 7.**
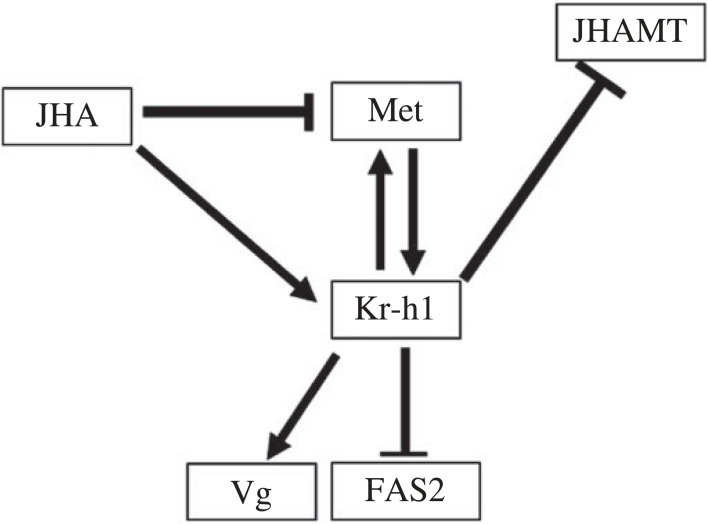
Regulation of genes related to juvenile hormone signalling pathway in *T. absoluta.*

Based on the above results, we obtained the JH signalling cascade regulatory pattern shown in figure 7.

## 4. Discussion

The corpora allata (CA) of insects synthesizes and secretes JHs, which are sesquiterpenoids, regulating the growth and development of larvae, prevent premature puberty, stimulate female adults to promote ovarian maturation and yolk formation, and affect the appearance, moulting and diapause of larvae. As transcription factors and receptors of JH, *Takr-h1* and *TaMet* play a key role in the growth and development of tomato leaf miners. Therefore, we analysed the sequence structure of these two key genes and conducted a diversity analysis. Analysis of Takr-h1's conservative domain and three-dimensional structure found that it contains eight Cys2/His2 (C2H2) type zinc finger structures (each consisting of 22–25 amino acids that regulate gene expression at both the transcriptional and translational levels) (electronic supplementary material, figure S2*c*/*e*), which are involved in biological processes such as development, recombination and chromatin regulation [59–61]. Specifically, DNA-binding zinc finger proteins bind DNA through multiple closely connected zinc finger domains in series, with each individual zinc finger structure binding to a continuous three-nucleotide subsequence and forming a potential fourth cross strand contact, which overlaps with the target gene of the N-terminal adjacent zinc finger structure [62]. In arthropods, a considerable portion of C2H2 proteins contain an N-terminal ZAD domain (zinc finger-associated domain) responsible for homodimerization [63,64]. Krüppel-like transcription factors (KLF) can interact with GATA proteins and zinc finger proteins of other families, enabling it to regulate gene expression and the interactions between nucleic acids and proteins [65]. According to multiple sequence alignment, the similarity of Takr-h1's amino acid sequence to other insects was 85.27% (electronic supplementary material, figure S2*e*), indicating the gene is highly conserved across insects. The domain and three-dimensional structure predictions for TaMet protein showed that it belongs to the bHLH-PAS protein family, meaning it TaMet contains a bHLH domain, PAS domain and PAC domain (electronic supplementary material, figure S2*d*). As a transcription regulator, TaMet protein plays an important role in regulation of growth, development and physiology [66]. Typically, a bHLH domain contains approximately 60 amino acid residues and can be divided into two functional regions: approximately 15 amino acids comprise the basic region responsible for DNA binding, and the region near the C-terminal is involved in protein dimerization [67]. In the TaMet protein, the bHLH region comprises 56 amino acids, and the two PAS domains contain 67 and 69 amino acids, respectively, and are separated by a poorly conserved spacer (electronic supplementary material, figure S2*f*). The PAS domain can mediate various biochemical functions: dimerization between PAS proteins, small molecule binding and non-PAS protein interactions [68–70]. These two regions are usually linked to a PAS-associated C-terminal (PAC) motif [71].

*Takr-h1* is the transcription factor of JH, whereas *TaMet* is the receptor protein of JH. Therefore, both respond to JH and regulate the growth and development of insects through both their unique and conserved domains. These domains provide possible directions for finding target genes to control unwanted growth of tomato leaf miners. Notably, Met receptors are found in many insects, such as *T. castaneum*, *D. melanogaster* and *Aedes aegypti* [72]. Through multiple sequence comparison with other species, it was found that the Met protein sequence is also highly conserved in insects. Studies have shown that the amino acid sequences of GmMet and GmKr-h1 are highly homologous to those in other lepidopteran insects, especially *H. armigera*. Furthermore, these studies show that JH's functions in development, metamorphosis and reproduction are conserved in the evolutions of both complete and incomplete metamorphosis [20,35]. Therefore, the JH signalling pathway presents an appropriate target site for pest control with dsRNA. In particular, the critical role of JH in insect development and reproduction has motivated researchers to investigate JH signalling, and here we explore the functional role of the related genes based on their temporal expression patterns. The data in our study showed that there was no significant difference in the relative expression level of *TaMet* in the larval stage. However, the expression level increased significantly during the late pupal stage. The low relative expression of *TaMet* in larval stage helps larvae enter the next stage and complete pupation. At the late pupal stage, the expression of *TaMet* gene increased significantly, which further indicated that *TaMet* gene plays a regulatory role in metamorphosis. Studies with *Chilo suppressalis* showed that the expression level of *CsMet1* decreased significantly during the prepupal stage, ensuring the insect's metamorphosis from larvae to pupa [73]. *HaMet1* of *H. armigera* and *SmMet* of *Sitodiplosis mosellana* exhibit similar phenomena, confirming Met acts as a receptor to play an anti-metamorphic role in metamorphosis development [19,74,75].

*Kr-h1* is an early JH response gene in many insects, and its JH-induced expression depends on the function of Met [11,13,76–78]. The relative expression level of *Takr-h1* in the larval stage and the early pupal stage was significantly lower than that in the late pupal stage, and the expression level in the newly emerged males was significantly higher than that in the females (figure 2). Declined *S. mosellana kr-h1* expression level in the pre-pupae stage is related to the larval-pupal metamorphosis [79], so we speculated that the low relative expression level of *Takr-h1* in the stage of larval and early pupa is conducive to the metamorphosis and development of tomato leaf miners. In the expression profile of *TaFOXO*, we found that expression of *TaFOXO* increased significantly from the pupa stage to male eclosion (figure 2). Related studies found that the expression of *FOXO* was elevated during moulting and metamorphosis in *H. armigera*, proposing the idea that this gene is a key regulator of protein hydrolysis induced by 20E [80]. Similarly, the relative expression level of *TaJHAMT* in the pupal stage was extremely low, but increase significantly during male eclosion (figure 2). In the study of *A. aegypti*, the transcript levels of *AaJHAMT* were almost undetectable in the CA of freshly pupated and 1-day-old pupae; however, *AaJHAMT* mRNA expression levels were significantly higher in newly emerged females [81]. Transcription inhibition of *BmJHAMT* gene is essential for the termination of JH biosynthesis in CA, which is a prerequisite for the initiation of metamorphosis [51].

During the metamorphosis of *D. melanogaster*, JH induces the expression of *kr-h1* through Met and Gce receptors, thereby maintaining larval morphology and regulating larval metamorphosis and development [82]. In a study with *Nilaparvata lugens*, it was shown that deletion of the *Nlkr-h1* gene resulted in the production of a nymph-adult intermediate with incompletely extended membrane wings and deformed external genitalia [83]. Additionally, interfering with *Met* expression in *T. castaneum* led to the smaller pupae, larvae and pupal intermediates [72]. Another study with *Bactrocera dorsalis* confirmed that *kr-h1* and *Met* were involved in vitellogenesis and oviposition [84], thereby indicating the role of JH signalling pathway in insect reproduction. Taken together, these studies reflect the gradual development of the *kr-h1-* and *Met-* regulating model for JH, and our current study shows that *Takr-h1* is indeed involved in the growth and development of tomato leaf miners. In this study, we effectively suppressed expression of *Takr-h1*, and in doing so, found that the mortality of the second instar larvae increased compared with the control group (figure 3). Even if some larvae still entered the pupal stage, there was a significant risk of deformity (figure 4). Therefore, our experiments confirmed that the gene has a key role in regulating the growth, development and metamorphosis of larvae. Furthermore, the expression of *GmMet* and *Gmkr-h1* were both inhibited by dsRNA injection, resulting in reduced larval survival, pupae malformation, and decreased fecundity of surviving adults [20]. This prompted us to explore the regulatory effect between *Met* and *kr-h1* in greater depth.

Notably, *TaMet* expression of was significantly diminished following RNAi of *Takr-h1* (figure 5). Similarly, the relative expression of *Takr-h1* decreased significantly in response to *TaMet* interference (figure 6). This phenomenon indicates that there is a positive regulatory relationship between the two genes. With the identification of the *Met* and *kr-h1* factors, the *Met*-*kr-h1* regulatory model has been reported in detail among metamorphosed insects [85], with these findings crucially strengthening our understanding of the key genes involved in the JH pathway. Interestingly, JHA can induce *kr-h1* expression in the second instar larvae of tomato leaf miner, and this same effect has been verified in *Harmonia axyridis* [86] and *Geleruca daurica* [87]. The results of a study with *S. mosellana* showed that, following JHA treatment, the *SmMet* and *Smkr-h1* genes exhibited similar expression patterns, with their relative expression levels undergoing significant increases [79]. In another study, JH-II was injected into the body of *A. ypsilon*, and, consequently, it was found that the expression levels of the *Met1*, *Met2* and *kr-h1* genes increased significantly [88]. Unlike previous studies, in our study, tomato leaf miner expression of *TaMet* was inhibited after JHA treatment. This may be due to various factors, such as the difference in insects, periods, and response time to JHA, perhaps the expression of *TaMet* is only temporarily reduced. In another study, RNAi-mediated depletion of *Bmkr-h1* in female *B. mori* pupae suppressed *BmVg* expression and reduced Vg protein deposition in oocytes [89]. Similarly, applying RNAi to the tomato leaf miner, we found that the reduction of *Takr-h1* gene also inhibited the expression of *TaVg*, indicating that there was a positive regulatory relationship between the two. As JH synthesis pathway enzyme in the that converts JH acid or inactive JH precursors into active JHs, JHAMT plays a key role in regulating JH synthesis and, in turn, exerts effects on insect growth, development, reproduction, diapause and polyphenisms process [51]. In this study with tomato leaf miners, the *TaJHAMT* expression pattern was similar to that of *Takr-h1*, where the expression levels were significantly lower and higher in larvae and newly emerging males, respectively (figure 2). With the decrease in *Takr-h1* gene expression, the expression of *TaJHAMT* increased significantly, indicating a negative regulatory relationship between them. Previous study has suggested that when RNAi of the JH acid methyltransferase (*TfMT3*) reduces JH, the imbalance between low JH and other unknown factors (i.e. including *TfKr-h1*) leads to prepupal arrest in flour beetles [90], indicating that the stable regulation between *JHAMT* and *kr-h1* is very important in the development of insects. Similarly, there is a negative regulatory relationship between *TaFAS2* and *Takr-h1*. Likewise, studies have shown that lack of *Met* induces *FAS2* expression, thereby promoting lipid storage in diapausing female beetles [54].

## 5. Conclusion

In summary, we identified *Takr-h1* as a target gene in the JH pathway that can be used for the control of tomato leaf miners. Specifically, interference of *Takr-h1* leads to increased larval mortality and pupae malformation, thereby providing a target for the research and development of green dsRNA preparation. In this study, the regulatory relationships between *Takr-h1*, *TaMet*, *TaVg*, *TaJHAMT* and *TaFAS2* in the JH pathway were also determined. According to these results, we propose the following regulatory model. (1) There is a positive regulatory relationship between *Takr-h1* and *TaMet*. (2) *Takr-h1* can activate the expression of *TaVg* and inhibit the expression of *TaJHAMT* and *TaFAS2*. (3) Exogenous JHA can promote the expression of *Takr-h1* and inhibit the expression of *TaMet*. In summary, our study is the first to identify the key factors in the JH pathway of a major invasive pest: the tomato leaf miner. Furthermore, we conducted a preliminary exploration of the regulatory relationships within this pathway, thereby providing a basis for subsequent research.

## Data Availability

The DNA sequences obtained in this study have been deposited in the GenBank with accession codes OQ615790 (*Takr-h1*), OQ615791 (*TaMet*), OQ615792 (*TaFOXO*), OQ615793 (*TaJHAMT*), OQ615794 (*TaJHE*) and OQ615795 (*TaFAS2*). The data are provided in electronic supplementary material [91].
